# DNA Barcode Identification of Freshwater Snails in the Family Bithyniidae from Thailand

**DOI:** 10.1371/journal.pone.0079144

**Published:** 2013-11-04

**Authors:** Jutharat Kulsantiwong, Sattrachai Prasopdee, Jiraporn Ruangsittichai, Wipaporn Ruangjirachuporn, Thidarut Boonmars, Vithoon Viyanant, Paola Pierossi, Paul D. N. Hebert, Smarn Tesana

**Affiliations:** 1 Food-Borne Parasite Research Group, Department of Parasitology, Faculty of Medicine, Khon Kaen University, Khon Kaen, Thailand; 2 Department of Medical Entomology, Faculty of Tropical Medicine, Mahidol University, Bangkok, Thailand; 3 Center of Excellence for Research in Biomedical Sciences, and Thailand Center of Excellence on Drug Discovery and Development, Thammasat University, Klongluang, Pathumthani, Thailand; 4 Biodiversity Institute of Ontario, University of Guelph, Guelph, Ontario, Canada; Australian Museum, Australia

## Abstract

Freshwater snails in the family Bithyniidae are the first intermediate host for Southeast Asian liver fluke (*Opisthorchis viverrini*), the causative agent of opisthorchiasis. Unfortunately, the subtle morphological characters that differentiate species in this group are not easily discerned by non-specialists. This is a serious matter because the identification of bithyniid species is a fundamental prerequisite for better understanding of the epidemiology of this disease. Because DNA barcoding, the analysis of sequence diversity in the 5’ region of the mitochondrial COI gene, has shown strong performance in other taxonomic groups, we decided to test its capacity to resolve 10 species/ subspecies of bithyniids from Thailand. Our analysis of 217 specimens indicated that COI sequences delivered species-level identification for 9 of 10 currently recognized species. The mean intraspecific divergence of COI was 2.3% (range 0-9.2 %), whereas sequence divergences between congeneric species averaged 8.7% (range 0-22.2 %). Although our results indicate that DNA barcoding can differentiate species of these medically-important snails, we also detected evidence for the presence of one overlooked species and one possible case of synonymy.

## Introduction

Molecular taxonomic methods have been used extensively to complement morphological approaches for species identification, and for establishing phylogenetic relationships [1-10]. Particularly, species identification through DNA barcoding has seen rapid adoption over the past decade. Prior DNA barcode studies have clearly established their effectiveness in the delimitation of animal species, and also contributed several advantages [11-13]. The ability of DNA barcoding to identify all life stages has particular importance in medical parasitology, where it is not only important to identify the parasite and its final host, but also all its life stages and its intermediate hosts. Thus, a multidisciplinary method of classification that includes morphological, molecular and distributional data is an essential prerequisite for understanding the epidemiology of any parasite-induced disease [7]. 

Freshwater snails of the family Bithyniidae serve as intermediate hosts for the liver fluke, *Opisthorchis viverrini*, and related species common in the Greater Mekong subregion (Cambodia, Lao People’s Democratic Republic, Vietnam, and Thailand). The infection of this parasite has been associated with several hepatobiliary diseases, including opisthorchiasis, cholangitis, obstructive jaundice, hepatomegaly, cholecystitis, and biliary lithiasis [14-18]. Furthermore, both experimental and epidemiological evidence suggest that liver fluke infections can be an etiological factor of cholangiocarcinoma [19-25]. Three taxa of *Bithynia* are involved in the transmission of this parasite [26-28] with different species reported as intermediate hosts in different parts of Thailand. *B. siamensis goniomphalos* is a dominant host in the northeast, while *B. funiculata* and *B. siamensis siamensis* serve as hosts in the north and *B. siamensis siamensis* in the central region [26,29]. Taxonomic keys for differentiation to species in the family Bithyniidae utilized size, shape, color, and sculpture on the shell surface, operculum structure, and shape and arrangement patterns of radular teeth. Because these characters often demonstrate both geographic variation and phenotypic plasticity, morphological characters used to separate species are difficult to score and identifications require expert malacologists [30]. DNA barcoding has effectively identified snail species in other settings [31-34], therefore we decided to test its effectiveness on Bithyniidae.

The present study is the first to explore the application of DNA barcoding in species identification in the family Bithyniidae. We analyzed variation of the COI barcode region within 10 species/subspecies of Bithyniidae using pairwise sequence comparisons. We then examined the effectiveness of DNA barcoding in differentiating among these species. 

## Materials and Methods

### Snail collections and preparation

**Figure 1:**
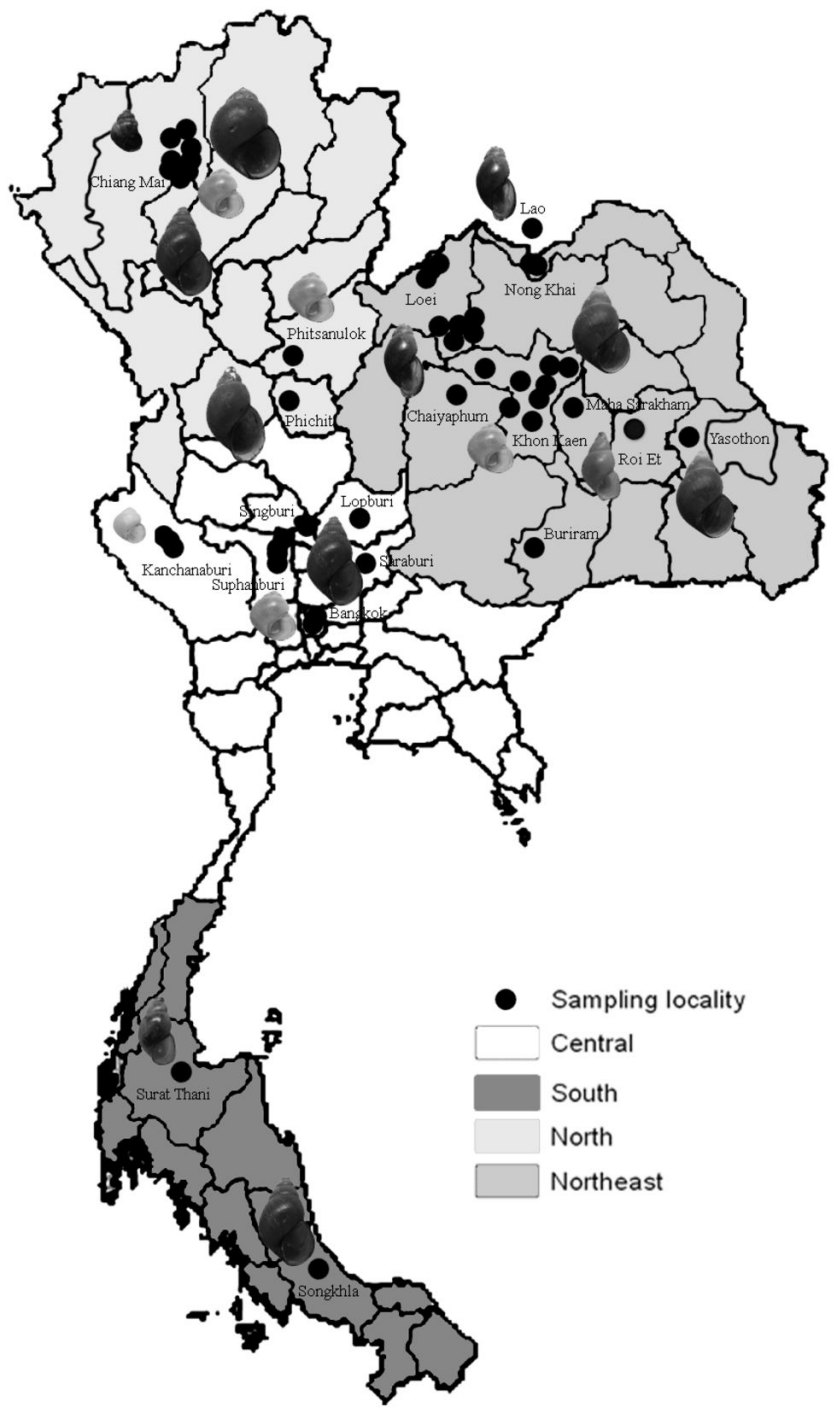
Schematic map of Thailand showing collection localities.

 Adult snails of the family Bithyniidae (superfamily Rissoacea) were collected with wire-mesh scoops or by hand in 2009 and 2010 from four regions of Thailand: north, northeast, south, and central (Figure 1, Table 1). These regions were selected based on results from previous studies [26,28,35]. Each collection site was recorded and its GPS coordinates were determined using a Garmin^®^nuvi 203 (Garmin (Asia) Co.,Taiwan). The specimens for this study were collected mostly from public water reservoirs where no permits were required. Owners of the private localities (a rice paddy and a waterfall) were asked for their permission. The owners gave their verbal consent for samples to be collected. All species of those snails are not endangered or protected. The snails were sorted and identified following the protocols in Brandt [26], Chitramvong [36], and Upatham et al. [37]. In addition two subspecies (*B*. *s. siamensis* and *B. s. goniomphalos*) were categorized by geographic distribution.

**Table 1:** Collection sites for each species from Thailand.

**Species**	**Collection Date**	**Country**	**State/Province**	Region^*^	**Latitude**	**Longitude**
*Bithynia funiculata*	09-May-2009	Thailand	Chiang Mai	Mae Rim1	18.68280029	98.97660065
(Walker, 1927)	09-May-2009	Thailand	Chiang Mai	Mae Rim2	18.91769981	98.97409821
	09-May-2009	Thailand	Chiang Mai	Saraphi3	18.68889999	98.9536972
	09-May-2009	Thailand	Chiang Mai	Mae Rim4	18.91139984	98.96800232
*Bithynia siamensis*	13-Oct-2010	Thailand	Nong Khai	Sangkhom5	18.09830093	102.2419968
*goniomphalos*	13-Oct-2010	Thailand	Nong Khai	Tha Bo6	17.78840065	102.6009979
(Morelet, 1866)	04-Jun-2010	Thailand	Roi Et	Muang Roi Et7	15.90060043	103.7320023
	03-May-2008	Thailand	Maha Sarakham	Barabue8	16.03829956	103.1190033
	11-May-2008	Thailand	Khon Kaen	Chum Phae9	16.54809952	102.0940018
	04-Apr-2008	Thailand	Khon Kaen	Ubolratana10	16.75279999	102.6330032
	10-May-2008	Thailand	Nong Bua Lamphu	Non Sang11	16.86380005	102.5680008
	04-Apr-2008	Thailand	Khon Kaen	Ban Phai12	16.16609955	102.6829987
	04-Apr-2008	Thailand	Khon Kaen	Waeng Noi13	15.80589962	102.4110031
	09-Dec-2008	Thailand	Khon Kaen	Ubolratana14	16.75300026	102.6330032
	04-Apr-2008	Thailand	Khon Kaen	Ban Phai15	16.16609955	102.6829987
	10-May-2010	Thailand	Buriram	Nong Ki16	14.66600037	102.5439987
	12-May-2008	Thailand	Khon Kaen	Muang Khon Kaen17	16.44829941	102.8499985
*Bithynia siamensis siamensis*	26-Feb-2011	Thailand	Songkhla	Hat Yai18	7.013070107	100.4520035
(Morelet, 1866)	10-Oct-2009	Thailand	Khon Kaen	Muang Khon Kaen19	16.44799995	102.8499985
	10-May-2009	Thailand	Bangkok	Kasertsat University20	13.85270023	100.5699997
	10-May-2009	Thailand	Bangkok	Kasertsat University21	13.8494997	100.5680008
	08-May-2009	Thailand	Phitsanulok	Bang Rakam22	16.67480087	100.1600037
	08-May-2009	Thailand	Phichit	Bueng Na Rang23	16.17670059	100.1279984
	09-May-2009	Thailand	Chiang Mai	Muang Chiang Mai24	18.80529976	98.95020294
	09-May-2009	Thailand	Chiang Mai	Muang Chiang Mai25	18.79179955	98.94629669
	10-May-2009	Thailand	Suphan Buri	Si Pranchan26	14.6697998	100.1159973
	10-Jun-2010	Thailand	Lop Buri	Chai Badan^27^	15.20429993	101.137001
	26-Feb-2011	Thailand	Songkhla	Hat Yai^28^	7.013070107	100.4520035
	10-May-2009	Thailand	Sing Buri	Muang Sing Buri^29^	14.8604002	100.3939972
**Species**	**Collection Date**	**Country**	**State/Province**	Region^*^	**Latitude**	**Longitude**
*Bithynia siamensis siamensis*	10-Jun-2010	Thailand	Lop Buri	Patthana Nikhom^30^	14.85579967	100.9899979
(Morelet, 1866)						
*Filopaludina*	10-Aug-2010	Thailand	Khon Kaen	Khon Kaen	16.46800041	102.8310013
*martensi martensi*				University^31^		
*Gabbia erawanensis*	11-May-2009	Thailand	Kanchanaburi	Erawan^32^	14.36789989	99.14369965
(Prayoonhong, Chitramvong&	17-May-2009	Thailand	Kanchanaburi	Erawan^33^	14.36800003	99.14399719
Upatham 1990)	11-May-2009	Thailand	Kanchanaburi	Erawan^34^	14.3689003	99.145401
*Gabbia pygmaea*	09-May-2009	Thailand	Chiang Mai	Mae Rim^35^	18.91139984	98.96800232
(Preston, 1908)						
*Gabbia wykoffi*	09-May-2009	Thailand	Chiang Mai	Saraphi^36^	18.68560028	99.04979706
(Brandt 1968)	04-Apr-2010	Thailand	Loei	Chiang Khan^37^	17.90600014	101.6880035
	04-Apr-2010	Thailand	Loei	Chiang Khan^38^	17.89599991	101.6699982
	04-Apr-2010	Thailand	Loei	Chiang Khan^39^	17.89489937	101.6709976
	12-Oct-2009	Thailand	Saraburi	Muang Saraburi^40^	14.53129959	100.9260025
	10-May-2009	Thailand	Suphan Buri	Muang Sing Buri^41^	14.85379982	100.3779984
	09-May-2009	Thailand	Chiang Mai	Saraphi^42^	18.68560028	99.04979706
	09-May-2009	Thailand	Chiang Mai	Hang Dong^43^	18.68889999	98.9536972
	09-May-2009	Thailand	Chiang Mai	Hang Dong^44^	18.68280029	98.97660065
	09-May-2009	Thailand	Bangkok	Kasertsat University^45^	13.8494997	100.5680008
	20-Oct-2009	Thailand	Khon Kaen	Ubolratana^46^	16.75279999	102.6330032
	20-Oct-2009	Thailand	Khon Kaen	Muang Khon Kaen^47^	16.45019913	103.0270004
	10-Aug-2009	Thailand	Chaiyaphum	Chatturat^48^	15.56820011	101.8430023
	10-May-2009	Thailand	Bangkok	Kasertsat University^49^	13.8494997	100.5680008
	11-May-2009	Thailand	Kanchanaburi	Erawan^50^	14.36789989	99.14369965
	20-Oct-2009	Thailand	Khon Kaen	Muang Khon Kaen^51^	16.45019913	103.0270004
	10-May-2010	Thailand	Loei	Chiang Khan^52^	17.90600014	101.6880035
	10-May-2010	Thailand	Loei	Chiang Khan^53^	17.8946991	101.6699982
	11-May-2008	Thailand	Khon Kaen	Ubolratana^54^	16.75279999	102.6330032
**Species**	**Collection Date**	**Country**	**State/Province**	Region^*^	**Latitude**	**Longitude**
*Hydrobioides nassa*	18-Jan-2009	Thailand	Sing Buri	Muang Sing Buri^55^	14.86400032	100.3960037
(Theobald, 1865)	09-May-2009	Thailand	Lamphun	Muang Lamphun^56^	18.62989998	99.04989624
	08-May-2009	Thailand	Phichit	Bueng Na Rang^57^	16.17670059	100.1279984
	09-May-2009	Thailand	Chiang Mai	San Kamphaeng^58^	18.91139984	98.96800232
	10-May-2009	Thailand	Sing Buri	Khai Bang Rachan^59^	14.80000019	100.3089981
	10-May-2009	Thailand	Sing Buri	Muang Sing Buri^60^	14.91609955	100.3850021
	09-May-2009	Thailand	Chiang Mai	San Kamphaeng^61^	18.76029968	99.07859802
*Wattebledia baschi*	10-Oct-2010	Thailand	Surat Thani	Phunphin^62^	9.11400032	99.23000336
(Brandt 1968)	10-Oct-2009	Thailand	Surat Thani	Phunphin^63^	9.113780022	99.229599
*Wattebledia crosseana*	04-Apr-2010	Thailand	Loei	Chiang Khan^64^	17.89599991	101.6699982
(Wattebled 1886)	04-Apr-2010	Thailand	Loei	Chiang Khan^65^	17.89100075	101.6439972
	04-Apr-2010	Thailand	Loei	Pak Chom^66^	18.02479935	101.9000015
	10-May-2010	Thailand	Loei	Chiang Khan^67^	18.08709908	101.9520035
	25-Dec-2008	Thailand	Loei	Chiang Khan^68^	18.08699989	101.9520035
	11-May-2008	Thailand	Khon Kaen	Ubolratana^69^	16.75279999	102.6330032
	11-May-2008	Thailand	Nong Bua Lamphu	Muang Nong Bua Lum	17.24449921	102.5169983
				Phu^70^		
	12-Feb-2009	Laos	Vientiane	Pakse^71^	15.12049961	105.8130035
	04-Apr-2010	Thailand	Loei	Chiang Khan^72^	17.89599991	101.6699982
	25-Dec-2008	Thailand	Khon Kaen	Ubolratana^73^	16.75279999	102.6330032
	10-May-2010	Thailand	Nong Khai	Tha Bo^74^	17.78800011	102.6009979
*Wattebledia siamensis*	20-Jan-2008	Thailand	Khon Kaen	Muang Khon	16.4484005	102.8499985
(Moellendorff, 1902)				Kaen^75^		
	20-Jan-2008	Thailand	Khon Kaen	Ubolratana^76^	16.75279999	102.6330032
	20-Jan-2008	Thailand	Khon Kaen	Muang Khon	16.45319939	102.4530029
				Kaen^77^		

* represent the collection sites in the map

 Each snail was subsequently examined for trematode infections by testing for cercarial shedding twice within a week. Prior to cercarial shedding, the snails were cleaned with dechlorinated tap-water. Shedding was induced under 25 W electric light bulbs for 2 hours at room temperature during the day. For species that shed cercaria at night, black covers were used to achieve total darkness and snails were allowed to shed overnight. Uninfected snails were soaked in phosphate buffered saline (PBS) containing antibiotics (200 unit/ml of penicillin and 100 µg/ml of streptomycin) for 3 to 4 hours before extraction of DNA to ensure that bacterial contamination was minimized.

 Each snail was dissected to remove its soft body parts, and kept at -20 °C until further analysis. Each specimen was labeled, databased and imaged. All specimen records are in the project ‘JUT- Mitochondrial DNA barcodes identification for snail in family Bithyniidae in Thailand’ on BOLD, the Barcode of Life Data Systems [38].

### DNA extraction

Total genomic DNA was extracted from whole snail tissue using methods similar to those in Winnepenninckx et al. [39]. Snail tissue was first homogenized in lysis buffer (2% w/v Cetyltri-ammonium bromide; CTAB, 1.4 M NaCl, 0.2% v/v β-mercaptoethanol, 20 mM EDTA, 100 mM TrisHCl pH 8, 0.2 mg/ml proteinase K), and then incubated at 55 °C for 6 hours. Subsequently, proteins were precipitated using phenol/chloroform (1:1) once, followed by phenol/ chloroform/ isoamylalcohol (25:24:1), centrifuged at 13,000 g for 10 min (4 °C) twice, and finally washed with chloroform (1:1). The upper aqueous layer was removed, and DNA was precipitated in isopropanol (2:3 v/v), mixed gently by inverting the tube a few times, put on ice for 15 min, and then spun in a microcentrifuge at 13,000 g for 5 min. After centrifugation, the supernatant was discarded; the DNA pellets were washed in 75% absolute ethanol, and centrifuged at 13,000 g for 5 min. After air-drying, the DNA pellet was re-suspended in TE buffer (10 mM Tris, 1mM EDTA, pH 8.0) and stored at -20 °C until analysis. The DNA concentration and purity were estimated by spectrophotometer (NanoVue, GE Healthcare UK limited, Buckinghamshire, UK) at an absorbance of 260 and 280 nm wavelengths. The extracted genomic DNA was then diluted to a working concentration of 10 ng/µl. 

### Amplification and sequencing

PCR protocols followed those used by the Canadian Centre for DNA Barcoding [40], with slight modifications. The PCR reaction was performed on a GeneAmp® PCR System 9700 Thermo Cycler (Applied Biosystem, Foster City, CA). The partial mitochondrial COI gene was amplified using the primers shown in Table 2 [41,42] in a total reaction volume of 50 µl. The amplification reaction consisted of 10xPCR buffer for 5 µl, 10 mM dNTP for 0.25 µl, 50 mM MgCl_2_ for 2.5 µl, forward primer for 0.5 µl, reverse primer for 0.5 µl, Platinum Taq polymerase for 0.24 µl, H_2_O for 36.01 µl and template for 5 µl. Standard conditions for COI gene amplification included initial denaturation at 94 °C for 1 min, five cycles of 94 °C for 30 sec, annealing at 45-50 °C for 40 sec, and extension at 72 °C for 1 min, following by 30 to 35 cycles of 94 °C for 30 sec, 51 to 54 °C for 40 sec, and 72 °C for 1 min, with a final extension at 72 °C for 10 min, followed by an indefinite hold at 4 °C [43-45]. PCR products were visualized on a 1.5% agarose gel and the specific band was cut and its DNA purified and then sequenced in the Biochemistry Department, Faculty of Medicine, Khon Kaen University; Pacific Science Co. LTD (Bangkok, Thailand) and at the Biodiversity Institute of Ontario, Canada.

**Table 2:** Primers used for PCR amplification and sequencing [41,42].

**Primer name**	**Sequence**	**Forward or Reverse**
LCO1490	5′ GGTCAACAAATCATAAAGATATTGG 3′	Forward
HCO2198	5′ TAAACTTCAGGGTGACCAAAAAATCA 3′	Reverse
GasF1_t1	5′ TGTAAAACGACGGCCAGTTTTCAACAAACCATAARGATATTGG 3′	Forward
GasF2_t1	5′ TGTAAAACGACGGCCAGTATTCTACAAACCACAAAGACATCGG 3′	Forward
GasF3_t1	5′ TGTAAAACGACGGCCAGTTTTCWACWAATCATAAAGATATTGG 3′	Forward
GasR1_t1	5′ CAGGAAACAGCTATGACACTTCWGGRTGHCCRAARAATCARAA 3′	Reverse
MGasF1_t1	5′ TGTAAAACGACGGCCAGTATAAGATTTCCTCGWWTRAATAATA 3′	Forward
MGasR1_t1	5′ CAGGAAACAGCTATGACTCCTGTWCCWRCWCCWCCTTC 3′	Reverse

Remark Degenerate base; R = A or G, W = A or T, H = C or A or T

### Data analysis

Forward and reverse DNA sequences were assembled, and edited using Chromas version 2.23 [46], BioEdit v. 5.0.6 [47] and CodonCode v.3.01 (CodonCode Corporation, Dedham, MA). Alignment and homology analysis were performed using CLUSTAL X v. 1.8 [48] and MEGA 4 [49] with pairwise nucleotide sequence divergences calculated using the Kimura 2-parameter (K2P) model [50]. Base composition and distance summaries were obtained using the tools provided on the BOLD workbench (www.boldsystems.org) [38], but only sequences ≥ 350 bp were included in the analysis. A neighbour-joining (NJ) tree was also created using BOLD to provide a preliminary display of the sequence divergences. 

## Results and Discussion

**Figure 2:**
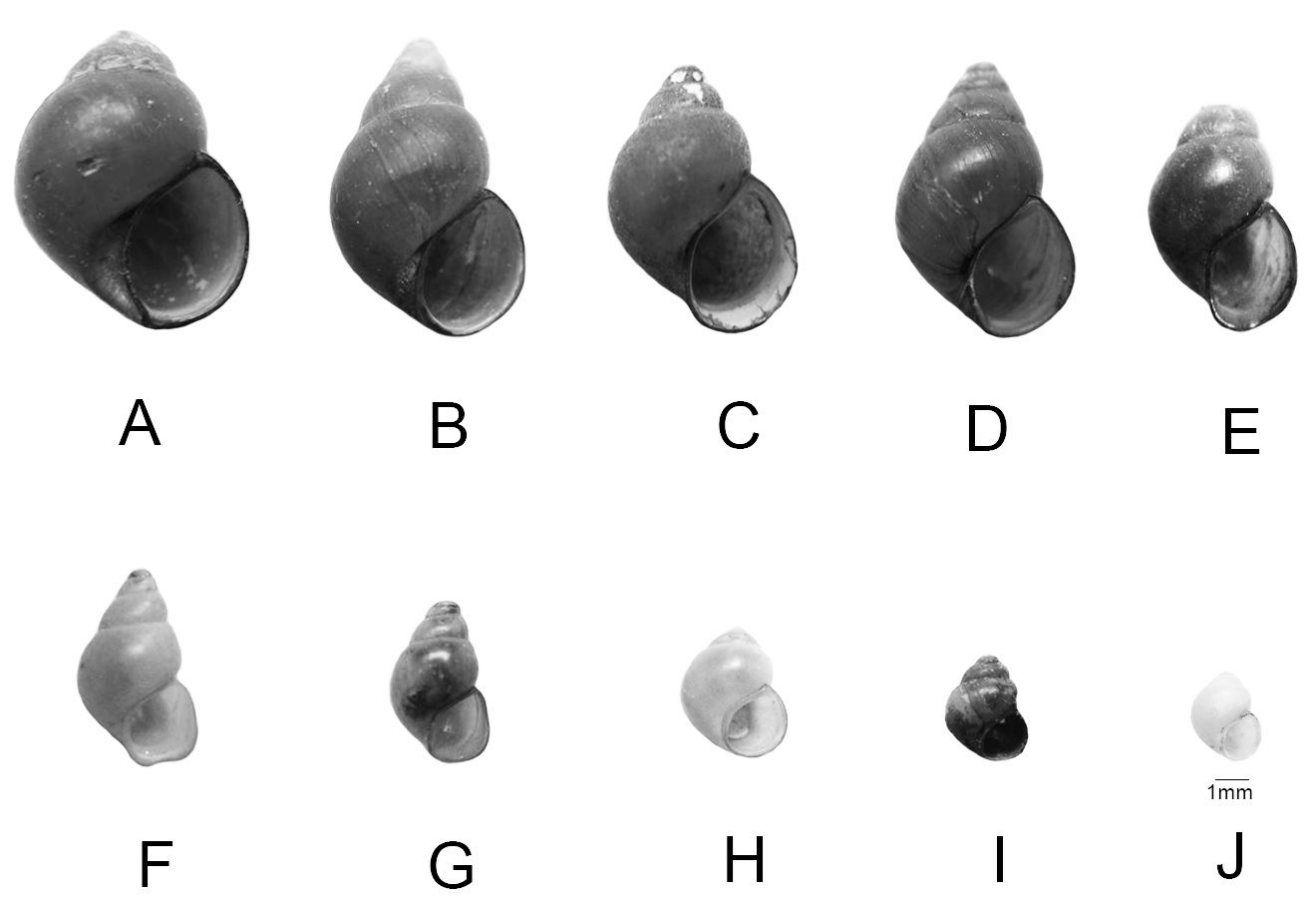
The shell morphology of bithyniid snails (A) *B. funiculata*; (B) *B. siamensis* *goniomphalos*; (C) *B. siamensis siamensis*; (D) *H. nassa* ; (E) *W. crosseana*; (F) *W. siamensis*; (G) *W. baschi*; (H) *G. wykoffi*; (I) *G. pygmaea*; (J) *G. erawanensis*. Scale bars: A-J = 1 mm.

Ten species/subspecies of Bithyniidae were collected from sites across Thailand (Figure 1 and Figure 2). A total of 217 individuals of these species/subspecies were analyzed for COI, and *Neotricula aperta* gamma strain (family Hydrobiidae, superfamily Rissoacea) from GenBank (Accession: AF AF188222.1 GI: 11493624 and AF188220.1 GI: 11493620) was used as outgroup. All 217 specimens were identified using morphological characteristics of the adult shells, radular patterns, geographic distribution [35-37], and confirmed by a malacologist. From 1-6 individuals of each species/subspecies from each of the five regions were analyzed, as shown in the neighbour-joining tree (Figure S1). The sequences, and trace files, are available on BOLD (project: JUT).

**Figure 3:**
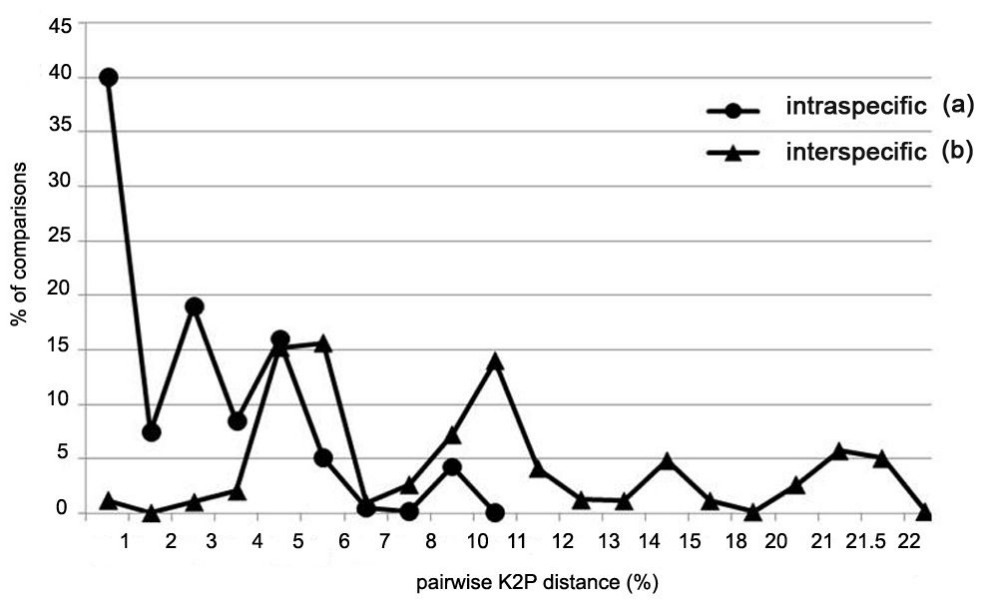
Pairwise distances (K2P) for COI sequences from snail species in the family Bithyniidae separated into two categories: (a) intraspecific; (b) interspecific.

The pairwise sequence divergences were different among species/subspecies (Table S1). Intraspecific K2P distances averaged 2.3±0.001% (range 0-9.2 %), 4-fold less than the mean congeneric sequence divergence of 8.7±0.002% (range 0-22.2 %). The highest mean intraspecific sequence divergence for an individual species was 4.93±0.22% (range 0-9.2%) for *Wattebledia crosseana* reflecting the fact that members of this species fell into two distinct sequence clusters (Table 3). The mean sequence divergence across the family was also high, averaging 17.1% (range 13.0-21.3%).The distributions of intraspecific and interspecific divergences showed limited overlap (Figure 3), because most (65.4%) intraspecific sequences showed less than 2% divergence while 83.4% of the interspecific sequences possessed more than 3% divergence. As a result, sequence divergences for these snails are similar to those in previous barcoding reports on other organisms [2,12]. Hebert et al. [12] reported that COI sequence divergences among animal species from interspecific COI divergences within the phylum Mollusca averaged 11.1±5.1%.

**Table 3:** Species with nearest neighbour and intraspecific and interspecific divergence.

**Species**	**Nearest Neighbor (NN)**		**Intraspecific**					**Intraspecific**				
**(Number of specimens)**												
	**Nearest Neighbor**	**Distance to**	**Count**	**Mean**	**SE**	**Max**	**Min**	**Count**	**Mean**	**SE**	**Max**	**Min**
		**NN%**	**Comparisons**					**Comparisons**				
*Bithynia funiculata* (13)	*B. siamensis siamensis*	7.11	78	1.08	0.10	2.17	0					
*B. siamensis goniomphalos* (30)	*B. siamensis siamensis*	1.49	435	2.39	0.04	3.95	0					
*B. siamensis siamensis* (40)	*B. siamensis goniomphalos*	1.49	780	0.62	0.50	1.81	0	2110	2.27	0.13	10.77	0
*Wattebledia crosseana* (26)	*W. baschi*	6.00	330	4.93	0.22	9.11	0					
*W. siamensis* (8)	*W. baschi*	11.39	28	0.32	0.44	0.82	0					
*W. baschi* (7)	*W. crosseana*	6.00	28	0.05	0.35	0.35	0	446	6.02	0.32	14.19	6.33
*Gabbia wykoffi* (59)	*G. pygmaea*	0	1761	3.14	0.05	6.69	0					
*G. pygmaea* (3)	*G. wykoffi*	0	3	0.00	0.00	0.00	0					
*G. erawanensis* (8)	*B. siamensis goniomphalos*	15.73	28	0.30	0.07	0.82	0	673	6.62	0.29	22.16	0
*Hydrobioides nassa* (23)	*W. crosseana*	14.13	253	0.47	0.03	2.20	0	0	0	0	0	0
**Total (217)**		**63.34**	**3724**	**13.30**	**1.81**	**27.91**	**0**	**3229**	**14.91**	**0.74**	**47.12**	**0**

The high intraspecific divergences in *W. crosseana* and *G. wykoffi* could indicate the presence of previously unrecognized cryptic species. DNA barcoding has proven invaluable at detecting cryptic species, which in many cases, are subsequently corroborated by life history, morphological or other character sets [51-54]. For these two snail species, the clusters represent allopatric populations with no apparent morphological differences, so it is currently unclear if they represent merely isolated populations or separate entities with differences yet to be revealed. Conversely, the sharing of identical barcode sequence in *G. pygmaea* and one northern Thailand population of *G. wykoffi* may be indicative of introgressive hybridization, incomplete lineage sorting, misidentification, or a previously unrecognized synonymy. Further investigations into these groups are necessary to untangle and confirm these predictions and the use of more holistic approaches to delimit species boundaries will be beneficial. 

An important finding in the present study is that the three first intermediate hosts (*B*. *s. siamensis, B. s. goniomphalos* and *B. funiculata*) of Southeast Asian liver fluke can all be distinguished by COI barcodes. All three taxa of *Bithynia* sp. form monophyletic clusters, with 1.5% divergence between the two subspecies of *B. siamensis* and both subspecies had 7.1% divergence from *B. funiculata* (Table 3). Because the two subspecies of *B. siamensis* are morphologically indistinguishable, the capacity of DNA barcoding to discriminate them is significant. Moreover, morphological similarity has created taxonomic confusion and difficulties in the accurate identification of *B*. *s. siamensis* and *B. s. goniomphalos* which are currently believed to be distributed in the north, central, south and northeast of Thailand [26,29,36-38]. As well, the capacity to rapidly diagnose all stages of the host’s life cycle is essential for better understanding of the epidemiology of this parasite-induced disease.

**Figure 4:**
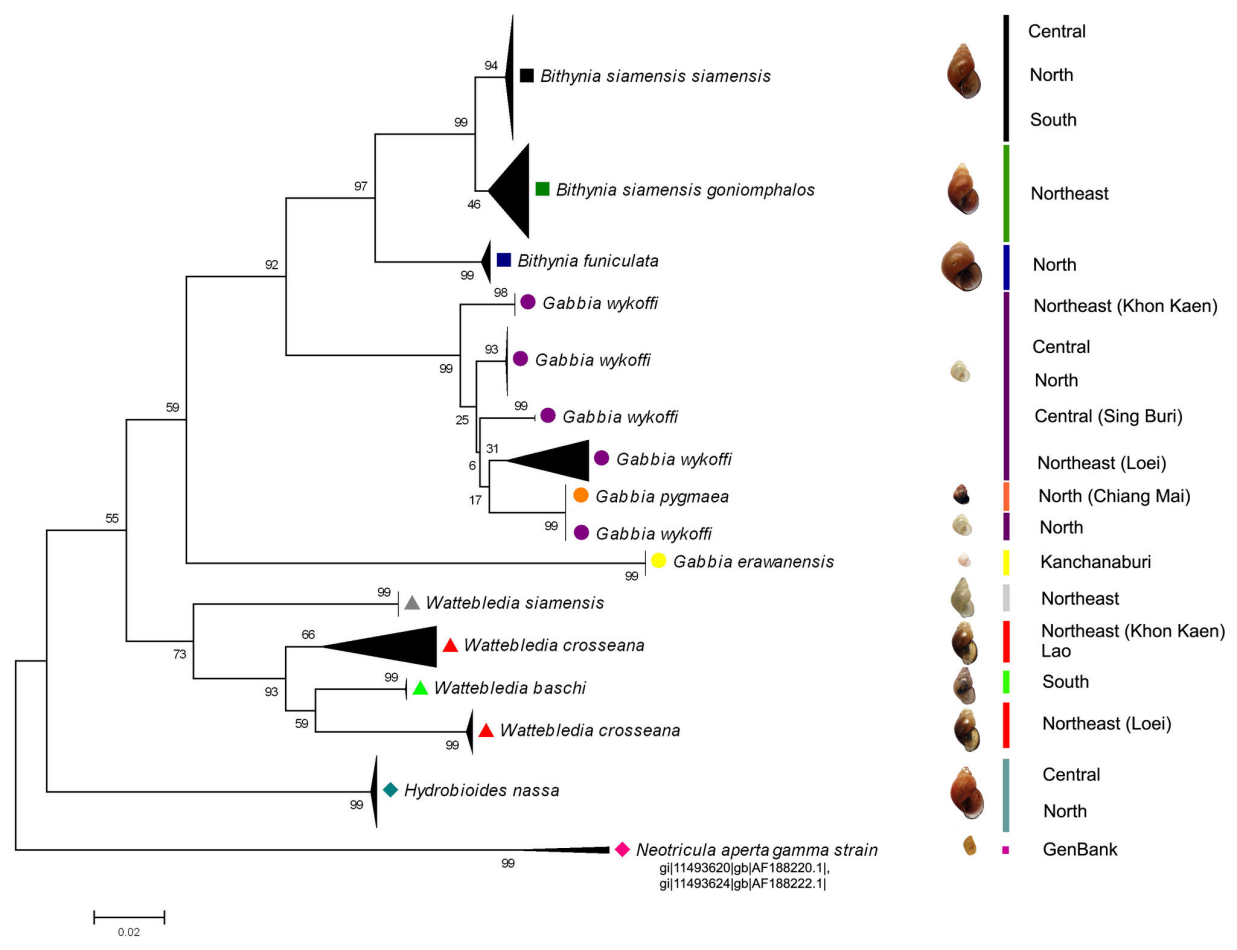
Neighbour- joining tree (K2P) for 10 species/subspecies of snails in the family Bithyniidae. The number of individuals for each branch is given in parentheses. A detailed version of this tree, including locality information, is provided in Figure S1.

 The barcoding success for the Bithyniid species examined in this project was 80%, with nearly all taxa forming discrete monophyletic clusters (Figure 4). The two exceptions are *G. pygmaea* and one population of *G. wykoffi*, which share an identical COI sequence (see above). These two taxa might possibly be cryptic species. However, the adult size of *G. wykoffi* is double that of *G. pygmaea*. Distinct paraphyly was found in *W. crosseana*. The results indicated that *W. crosseana* samples from different localities may well represent cryptic species because they are morphologically similar but genetically distinct. Cryptic species of *W. crosseana* might be resulted from some factors such as different localities which would develop to different genotype. *G. wykoffi* was separated into more than one geographically-restricted cluster respectively comprising collection localities from the central, north or northeast regions of Thailand. These clusters might be cryptic species according to this analysis as same *as W. crosseana*. However, more comprehensive analyses of the systematics of these taxa using more specimens, representing their known geographic distribution, as well as more evidence from independent biological investigations, are required before this hypothesis can be verified.

Similar studies which have also been reported in other organisms [52-59], yet over all DNA barcoding has proven reliable in identifying species in more than 90% of the organisms investigated [60]. The neighbour-joining tree and ME analysis also revealed that in general, individuals tended to cluster in accordance with collection localities (Supporting Information, Figure S1, S2). The results from ME analysis were very similar to the neighbor-joining analysis so the latter was used to generate diagrams. 

The genera *Wattebledia* and *Bithynia* formed monophyletic clusters as well, but *Gabbia* did not. The selection of *Neotricula aperta* gamma strain (in the same superfamily) from GenBank as the outgroup appeared legitimate as it clustered separately from other snails in family Bithyniidae. Increased taxon, geographic, and gene sampling would be worthwhile to further explore the two ‘barcode outliers’ and the ability of COI to infer geographic provenance and phylogenetic affinities in this group. 

In summary, the present study has studied genetic-variation in ten species/subspecies of Bithyniidae from Thailand using COI. Sequence divergences were lower for intraspecific than congeneric comparison. Using COI, 80% of the studied snail taxa could accurately identified. In comparison with other methods for identifying snails in this family, DNA barcoding is quicker, easier and more applicable, it is suitable for young snail identification which will be beneficial for understanding the epidemiology of opisthorchiasis transmission.

## Supporting Information

Table S1 **Genetic distances for all specimens in family Bithyniidae.**
(XLS)

Figure S1 **Neighbour-joining tree (Distance model: Kimura-2-Parameter) of profile and test taxa; includes a list of BOLD with Process ID, taxa names, length of sequence and locality.**
(JPG)

Figure S2 **Minimum Evolution tree (ME) of 218 COI sequences of 10 species/subspecies of snails in the family Bithyniidae.** The number of individuals for each branch is given in parentheses.
(TIF)

## References

[1] Thomas W, Davis GM, Chen CE, Zhou XN, Zeng PX et al. (2000) *Oncomelania* *hupensis* (Gastropoda: Rissooidea) in eastern China: molecular phylogeny, population structure, and ecology. Acta Trop 77: 215-227. doi:10.1016/S0001-706X(00)00143-1. PubMed: 11080513. 11080513

[2] Carvalho OS, Caldeira RL, Simpson AJG, THDA Vidigal (2001) Genetic variability and molecular identification of Brazilian *Biomphalaria* species (Mollusca: Planorbidae). Parasitology 123: S197-S209. PubMed: 11769284. 11769284 10.1017/s0031182001008058

[3] Jones CS, Rollinson D, Mimpfoundi R, Ouma J, Kariuki HC et al. (2001) Molecular evolution of freshwater snail intermediate hosts within the *Bulinus* *forskalii* group. Parasitology 7: 277-292. PubMed: 11769290. 10.1017/s0031182001008381 11769290

[4] Davis GM, Wilke T, Spolsky CM, Qiu CP, Qiu DC et al. (1998) Cytochrome oxidase I-based phylogenetic relationships among the Pomatiopsidae, Hydrobiidae, Rissoidae and Truncatellidae (Gastropoda: Caenogastropoda: Rissoacea). Malacologia 40: 251-266.

[5] Delicado D, Ramos MA (2012) Morphological and molecular evidence for cryptic species of springsnails [genus **Pseudamnicola** ( **Corrosella**) (Mollusca, Caenogastropoda, Hydrobiidae)]. Zookeys 190: 55-79. PubMed: 22639531. 10.3897/zookeys.190.2555 PMC3349067 22639531

[6] Kodcharin P (2006) Genetic variation of *Bithynia**siamensis**goniomphalos*, first intermediate host of *Opisthorchis**viverrini* in the basin of Mun and Chi River, Thailand by RAPD. M.Sc. Thesis: The Graduate School, Khon Kaen University, Khon Kaen, Thailand..

[7] Duangprompo W (2007) Genetic variation of snails in the family Bithyniidae in Thailand and identification of *Bithynia**siamensis**goniomphalos* by PCR. M.Sc. Thesis: The Graduate School, Khon Kaen University, Khon Kaen, Thailand..

[8] Jorgensen A, Kristensen TK, Stothard JR (2007) Phylogeny and biogeography of African *Biomphalaria* (Gastropoda: Planorbidae), with emphasis on endemic species of the Great East African lakes. Zool J Linn Soc 151: 337-349. doi:10.1111/j.1096-3642.2007.00330.x.

[9] Caldeira RL, Jannotti-Passos LK, Carvalho OS (2009) Molecular epidemiology of Brazilian *Biomphalaria*: A review of the identification of species and the detection of infected snails. Acta_Trop 111: 1-6. PubMed: 19426656. 19426656 10.1016/j.actatropica.2009.02.004

[10] Kiatsopit N, Sithithaworn P, Boonmars T, Tesana S, Chanawong A et al. (2011) Genetic markers for studies on the systematics and population genetics of snails, *Bithynia* spp., the first intermediate hosts of *Opisthorchis* *viverrini* in Thailand. Acta Trop 118: 136-141. doi:10.1016/j.actatropica.2011.02.002. PubMed: 21352793. 21352793

[11] Hebert PDN, Cywinska A, Ball SL, deWaard JR (2003) Biological identiﬁcations through DNA barcodes. Proc R Soc Lond B 270: 313-321. doi:10.1098/rspb.2002.2218. PMC1691236 12614582

[12] Hebert PDN, Ratnasingham S, deWaard JR (2003) Barcoding animal life: cytochrome *c* oxidase subunit 1 divergences among closely related species. Proc R Soc Lond B 270: 96-99. doi:10.1098/rsbl.2003.0025. PubMed: 12952648. PMC1698023 12952648

[13] Ferri E, Barbuto M, Bain O, Galimberti A, Uni S et al. (2009) Integrated taxonomy: traditional approach and DNA barcoding for the identification of filarioid worms and related parasites (Nematoda). Front Zool 6: 1-12. doi:10.1186/1742-9994-6-1. PubMed: 19128479. 19128479 PMC2657783

[14] Harinasuta T, Riganti M, Bunnag D (1984) *Opisthorchis* *viverrini* infection: pathogenesis and clinical features. Arzneimittel Forschung 34: 1167-1169. PubMed: 6542384. 6542384

[15] Osman M, Lausten SB, El-Sefi T, Boghdadi I, Rashed MY et al. (1998) Biliary parasites. Dig Surg 15: 287-296. doi:10.1159/000018640. PubMed: 9845601. 9845601

[16] Mairiang E, Mairiang P (2003) Clinical manifestation of opisthorchiasis and treatment. Acta Trop 88: 221-227. doi:10.1016/j.actatropica.2003.03.001. PubMed: 14611876. 14611876

[17] Sripa B, Leungwattanawanit S, Nitta T, Wongkham C, Bhudhisawasdi V et al. (2005) Establishment and characterization of an opisthorchiasis-associated cholangiocarcinoma cell line (KKU-100). World J Gastroenterol 11: 3392-3397. PubMed: 15948244. 15948244 10.3748/wjg.v11.i22.3392 PMC4315993

[18] Sripa B, Kaewkes S, Sithithaworn P, Mairiang E, Laha T et al. (2007) Liver fluke induces cholangiocarcinoma. PLOS Med 7: 1148-1155. PubMed: 17622191. 10.1371/journal.pmed.0040201 PMC1913093 17622191

[19] Thamavit W, Bhamarapravati N, Sahaphong S, Vajrasthira S, Angsubhakom S (1978) Effects of dimethylnitrosamine on induction of cholangiocarcinoma in *Opisthorchis* *viverrini* infected Syrian golden hamsters. Cancer Res 38: 4634-4639. PubMed: 214229. 214229

[20] Haswell-Elkins MR, Sithithaworn P, Elkins D (1992) *Opisthorchis* *viverrini* and cholangiocarcinoma in Northeast Thailand. Parasitol Today 8: 86-89. doi:10.1016/0169-4758(92)90241-S. PubMed: 15463578. 15463578

[21] IARC (1994) Infection with liver flukes (*Opisthorchis* *viverrini*, *Opisthorchis* *felineus* and *Clonorchis* *sinensis*). IARC Monogr Eval Carcinog Risks Hum 61: 121-175. PubMed: 7715069. 7715069 PMC7681589

[22] Sithithaworn P, Haswell-Elkins MR, Mairiang P, Satarug S, Mairiang E et al. (1994) Parasite-associated morbidity: liver fluke infection and bile duct cancer in northeast Thailand. Int J Parasitol 24: 833-843. doi:10.1016/0020-7519(94)90009-4. PubMed: 7982745. 7982745

[23] Vatanasapt V, Parkin DM, Sriamporn S (2000) Epidemiology of liver cancer in Thailand. In: Vatanasapt V Sripa B Liver cancer in Thailand: Epidemiology, diagnosis and control. Khon Kaen, Thailand: Siriphan Press pp. 3-6.

[24] Watanapa P, Watanapa WB (2002) Liver fluke-associated cholangiocarcinoma. Br J Surg 89: 962-970. doi:10.1046/j.1365-2168.2002.02143.x. PubMed: 12153620. 12153620

[25] Honjo S, Srivatanakul P, Sriplung H, Kikukawa H, Hanai S et al. (2005) Genetic and environmental determinants of risk for cholangiocarcinoma via *Opisthorchis* *viverrini* in a densely infested area in Nakhon Phanom, northeast Thailand. Int J Cancer 117: 854-860. doi:10.1002/ijc.21146. PubMed: 15957169. 15957169

[26] Brandt RAM (1974) The non-marine aquatic Mollusca of Thailand. Archiv Mollusken 105: 1-423.

[27] TROPMED Technical Group (1986) Snails of medical importance in Southeast Asia. Southeast Asian J Trop Med Public Health 17: 282-322. PubMed: 3787310. 3787310

[28] Sri-Aroon P, Butraporn P, Limsomboon J, Kerdpuech Y, Kaewpoolsri M et al. (2005) Freshwater mollusks of medical importance in Kalasin Province, Northeast Thailand. Southeast Asian J Trop Med Public Health 36: 653-657. PubMed: 16124433. 16124433

[29] Wykoff DE, Harinasuta C, Juttijudata P, Winn MM (1965) *Opisthorchis* *viverrini* in Thailand- the life cycle and comparison with *O*. *felineus* . J Parasitol 51: 207-214. doi:10.2307/3276083. PubMed: 14275209. 14275209

[30] Rollinson D, Stothard JR, Jones CS, Lockyer AE, de Souza CP et al. (1998) Molecular characterization of intermediate snail hosts and the search for resistance genes. Mem Inst Oswaldo Cruz 93: 111-116. doi:10.1590/S0074-02761998000100020. 9921331

[31] Miller KB, Alarie Y, Wolfe GW, Whiting MF (2005) Association of insect life stages using DNA sequences: the larvae of *Philodyte* *sumbrinus* (Motschulsky) (Coleoptera: Dytiscidae). Syst Entomol 30: 499-509. doi:10.1111/j.1365-3113.2005.00320.x.

[32] Rojo S, Stahls G, Perez-Banon C (2006) Testing molecular barcodes: invariant mitochondrial DNA sequences vs. the larval and adult morphology of West Palaearctic *Pandasyopthalmus* species (Diptera: Syrphidae: Paragini). Eur J Entomol 103: 443-458.

[33] Puillandre N, Strong EE, Bouchet P, Boisselier MC, Couloux A et al. (2009) Identifying gastropod spawn from DNA barcodes: possible but not yet practicable. Mol Ecol Resour 9: 1311-1321. doi:10.1111/j.1755-0998.2009.02576.x. PubMed: 21564902. 21564902

[34] Shufran KA, Puterka GJ (2011) DNA barcoding to identify all life stages of holocycliccereal aphids (Hemiptera: Aphididae) on wheat and other Poaceae. Ann Entomol Soc Am 104: 39-42. doi:10.1603/AN10129.

[35] Chitramvong YP, Upatham ES (1989) A new species of freshwater snail for Thailand (Prosobranchia: Bithyniidae). Walkerana 3: 179-186.

[36] Chitramvong YP (1991) The Bithyniidae (Gastropoda: Prosobanchia) of Thailand: comparative internal anatomy. Walkerana 5: 161-206.

[37] Chitramvong YP (1992) The Bithyniidae (Gastropoda: Prosobranchia) of Thailand: comparative external morphology. Malacol Rev 25: 21-38.

[38] Sri-Aroon P, Butraporn P, Limsoomboon J, Kaewpoolsri M, Chusongsang Y et al. (2007) Freshwater mollusks at designated areas in eleven provinces of Thailand according to the water resource development projects. Southeast Asian J Trop Med Public Health 38: 294-301. PubMed: 17539279. 17539279

[39] Upatham ES, Sornmani S, Kitikoon V, Lohachit C, Burch JB (1983) Identification key for the fresh-and brackish-water snails of Thailand. Malacol Rev 16: 107-132.

[40] Ratnasingham S, Hebert PDN (2007) BOLD: The Barcode of Life Data System. Retrieved onpublished at whilst December year 1111 from www.barcodinglife.org. Mol Ecol Notes 7: 355-364 10.1111/j.1471-8286.2007.01678.x PMC1890991 18784790

[41] Winnepenninckx B, Backeljau T, De Wachter R (1993) Extraction of high molecular weight DNA from mollusks. Trends Genet 9: 407. doi:10.1016/0168-9525(93)90102-N. PubMed: 8122306. 8122306

[42] Canadian Centre for DNA Barcoding (CCDB) (2008) Advancing species identification and discovery. Available: http://www.ccdb.ca/. Accessed 12 October 2008.

[43] Folmer O, Black M, Hoeh W, Lutz R, Vrijenhoek R (1994) DNA primers for amplification of mitochondrial cytochrome c oxidase subunit I from diverse metazoan invertebrates. Mol Mar Biol Biotechnol 3: 294-299. PubMed: 7881515. 7881515

[44] Ivanova NV, Zemlak TS, Hanner RH, Hebert PDN (2007) Universal primer cocktails for fish DNA barcoding. Mol Ecol Notes 7: 544–548. doi:10.1111/j.1471-8286.2007.01748.x.

[45] Ivanova NV, deWaard JR, Hajibabaei M, Hebert PDN (2005) Protocols for high volume DNA barcoding. Available: http://www.dnabarcoding.ca/. Accessed 5 November 2008.

[46] Ivanova N, Grainger C (2006) Pre-made frozen PCR and sequencing plates. CCDB Advances, Methods Release No. 4. Available: http://www.ccdb .ca/pa/ge/research/protocols/ccdb-advances. Accessed 30 Nov 2008

[47] Ivanova N, Grainger C, Hajibabaei M (2006) Increased DNA barcode recovery using Platinum ^®^ *Taq*. CCDB Advances, Methods Release. Retrieved onpublished at whilst December year 1111 from No.2Available:http//www.ccdb.ca. Retrieved onpublished at whilst December year 1111 from /pa/ge/research/protocols/ccdb-advances. Accessed 30 Nov 2008

[48] McCarthy C (2008) Chromas. Available: http://technelysium.com.au/. Accessed 3 November 2009.

[49] Hall T (2008) BioEdit Sequence Alignment Editor for Windows 95/98/NT/XP. Available: http://www.mbio.ncsu.edu/bioedit/bioedit.html. Accessed 3 Oct 2009

[50] Larkin MA, Blackshields G, Brown NP, Chenna R, McGettigan PA et al. (2007) Clustal W and Clustal X version 2.0. Bioinformatics 23: 2947-2948. doi:10.1093/bioinformatics/btm404. PubMed: 17846036. 17846036

[51] Tamura K, Dudley J, Nei M, Kumar S (2007) MEGA4: Molecular Evolutionary Genetics Analysis (MEGA) software version 4.0. Mol Biol Evol 24: 1596-1599. Available: http://www.megasoftware.net/. Accessed 3 November 2009. doi:10.1093/molbev/msm092. PubMed: 17488738. 17488738

[52] Kimura M (1980) A simple method for estimating evolutionary rates of base substitutions through comparative studies of nucleotide sequences. J Mol Evol 16: 111-120. doi:10.1007/BF01731581. PubMed: 7463489. 7463489

[53] Hebert PDN, Penton EH, Burns JM, Janzen DH, Hallwachs W (2004) Ten species in one: DNA barcoding reveals cryptic species in the neotropical skipper butterfly *Astraptes* *fulgerator* . Proc Natl Acad Sci U S A 101: 14812-14817. doi:10.1073/pnas.0406166101. PubMed: 15465915. 15465915 PMC522015

[54] Smith MA, Rodriguez JJ, Whitfield JB, Deans AR, Janzen DH et al. (2008) Extreme diversity of tropical parasitoid wasps exposed by iterative integration of natural history, DNA barcoding, morphology, and collections. Proc Natl Acad Sci U S A 105: 12359-12364. doi:10.1073/pnas.0805319105. PubMed: 18716001. 18716001 PMC2518452

[55] Gibbs J (2009) Integrative taxonomy identifies new (and old) species in the *Lasioglossum* (*Dialictus*) *tegulare* (Robertson) species group (Hymenoptera, Halictidae). Zootaxa 2032: 1-38.

[56] Locke SA, McLaughlin JD, Dayanandan S, Marcogliese DJ (2010) Diversity and specificity in *Diplostomum* spp. metacercariae in freshwater fishes revealed by cytochrome *c* oxidase I and internal transcriber spacer sequences. Int J Parasitol 40: 333-343. doi:10.1016/j.ijpara.2009.08.012. PubMed: 19737570. 19737570

[57] Meyer CP, Paulay G (2005) DNA barcoding: error rates based on comprehensive sampling. PLOS Biol 3: e422. doi:10.1371/journal.pbio.0030422. PubMed: 16336051. 16336051 PMC1287506

[58] Meier R, Shiyang K, Vaidya G, Ng PKL (2006) DNA barcoding and taxonomy in Diptera: a tale of high intraspecific variability and low identification success. Syst Biol 55: 715-728. doi:10.1080/10635150600969864. PubMed: 17060194. 17060194

[59] Whitworth TL, Dawson RD, Magalon H, Baudry E (2007) DNA barcoding cannot reliably identify species of the blowfly genus *Protocalliphora* (Diptera : Calliphoridae). Proc R Soc Lond B 274: 1731-1739. doi:10.1098/rspb.2007.0062. PMC2493573 17472911

[60] Linares MC, Soto-Calderón ID, Lees DC, Anthony NM (2009) High mitochondrial diversity in geographically widespread butterflies of Madagascar: A test of the DNA barcoding approach. Mol Phylogenet Evol 50: 485-495. doi:10.1016/j.ympev.2008.11.008. PubMed: 19056502. 19056502

