# Supplementary figures and images for: DNA Barcode Identification of Freshwater Snails in the Family Bithyniidae from Thailand
Source: PLoS One. 2013 Nov 4;8(11):e79144. doi: 10.1371/journal.pone.0079144 (PMC3817070; doi:10.1371/journal.pone.0079144)

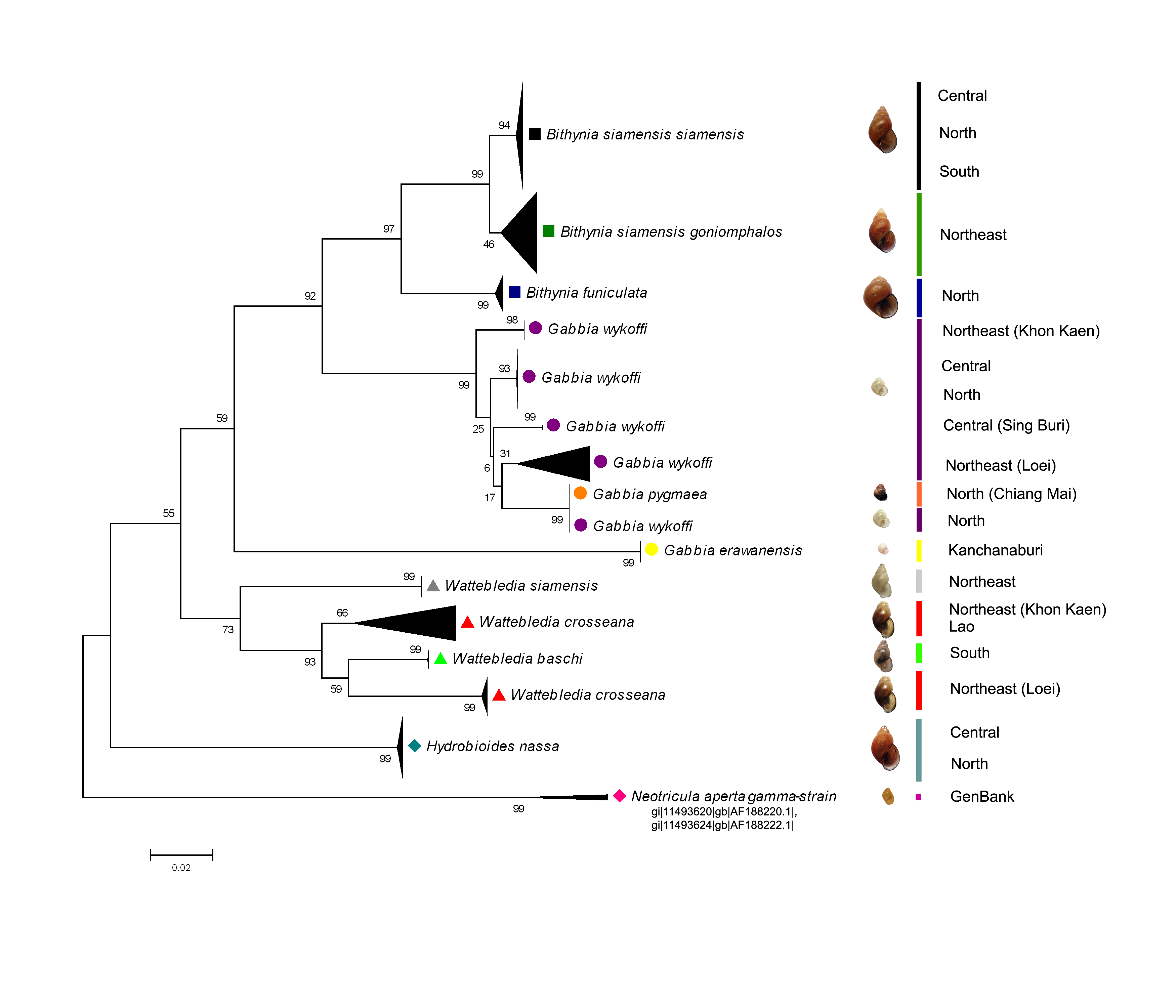

Supplement: Figure S2 — Minimum Evolution tree (ME) of 218 COI sequences of 10 species/subspecies of snails in the family Bithyniidae. The number of individuals for each branch is given in parentheses. (TIF) [file pone.0079144.s003.tif]
